# Association of COVID-19 With New Waiting List Registrations and Liver Transplantation for Alcoholic Hepatitis in the United States

**DOI:** 10.1001/jamanetworkopen.2021.31132

**Published:** 2021-10-26

**Authors:** Maia S. Anderson, Valeria S. M. Valbuena, Craig S. Brown, Seth A. Waits, Christopher J. Sonnenday, Michael Englesbe, Jessica L. Mellinger

**Affiliations:** 1 Department of Surgery, University of Michigan, Ann Arbor, Michigan; 2 Center for Healthcare Outcomes and Policy, University of Michigan, Ann Arbor, Michigan; 3 National Clinician Scholars Program, University of Michigan, Ann Arbor, Michigan; 4 Division of Gastroenterology and Hepatology, University of Michigan, Ann Arbor, Michigan

## Abstract

This cross-sectional study examines trends in new waiting list registrations and liver transplantation for alcoholic hepatitis before and during the COVID-19 pandemic in the US.

## Introduction

Alcohol consumption has substantially increased during the COVID-19 pandemic^1^; however, the impact on the already increasing burden of alcohol-associated liver disease (ALD) is unknown.^2^ We examined national changes in waiting list registration and liver transplantation for ALD and the association with alcohol sales during the COVID-19 pandemic. We hypothesized that waiting list registrations and deceased donor liver transplants (DDLTs) for alcoholic hepatitis (AH), which can develop after a short period of alcohol misuse, would disproportionately rise.

## Methods

This cross-sectional study was deemed exempt from review and informed consent by the University of Michigan institutional review board because the study involves deidentified, publicly available secondary data sets that cannot be tracked back to an individual. This study follows the Strengthening the Reporting of Observational Studies in Epidemiology (STROBE) reporting guideline for reporting cross-sectional studies.

Data were obtained from the United Network for Organ Sharing Standard Transplant Analysis and Research file for new waiting list registrations and primary DDLT for US adults (age ≥18 years) between January 1, 2016, and January 31, 2021, and categorized by primary listing diagnosis as AH, alcohol-related cirrhosis (AC), or non-ALD (any other diagnosis). For each diagnosis, we characterized short-term changes during COVID-19 (March 2020 to January 2021) by comparing proportions of waiting list registrations and DDLTs to the same time frame pre–COVID-19 (March 2019 to January 2020) using χ^2^ tests. To evaluate changes during COVID-19 compared with long-term trends, we compared observed monthly volumes of waiting list registrations and DDLTs from March 2020 to January 2021 with forecasted values based on pre–COVID-19 trends estimated from nonseasonal autoregressive integrated moving average models with first order differencing fit to monthly waiting list registrations and DDLTs for 4 years pre–COVID-19 (January 2016 to February 2020).^3^

National monthly retail alcohol sales from January 2016 to January 2021 for purchases from beer, wine, and liquor stores in US dollars were obtained from the US Census Bureau Monthly Retail Trade Report.^4^ The associations of alcohol sales, waiting list registrations, and DDLT were evaluated using Spearman rank-order correlation with a significance level of .05. Analyses were performed using Stata/SE version 16.0 (StataCorp). Data were analyzed from March to April 2021.

## Results

**Figure 1.**
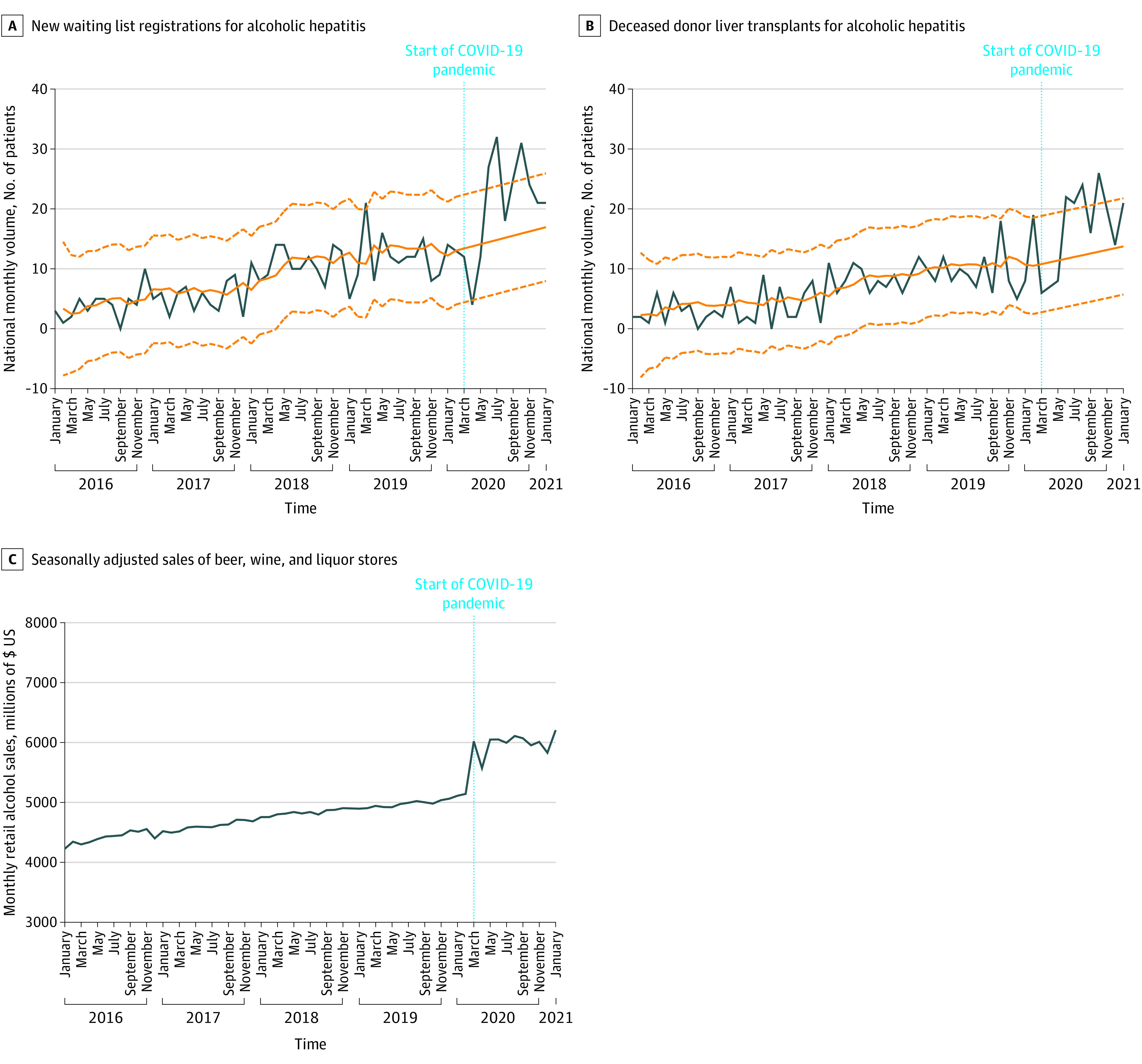
Observed and Forecasted National Monthly New Waiting List Registrations and Deceased Donor Liver Transplants for Alcoholic Hepatitis and Retail Alcohol Sales Before and During the COVID-19 Pandemic Observed values are shown in blue. Historical trends and forecasted values in the absence of COVID-19 (solid line) with 95% CIs (dashed lines) are shown in orange.

**Figure 2.**
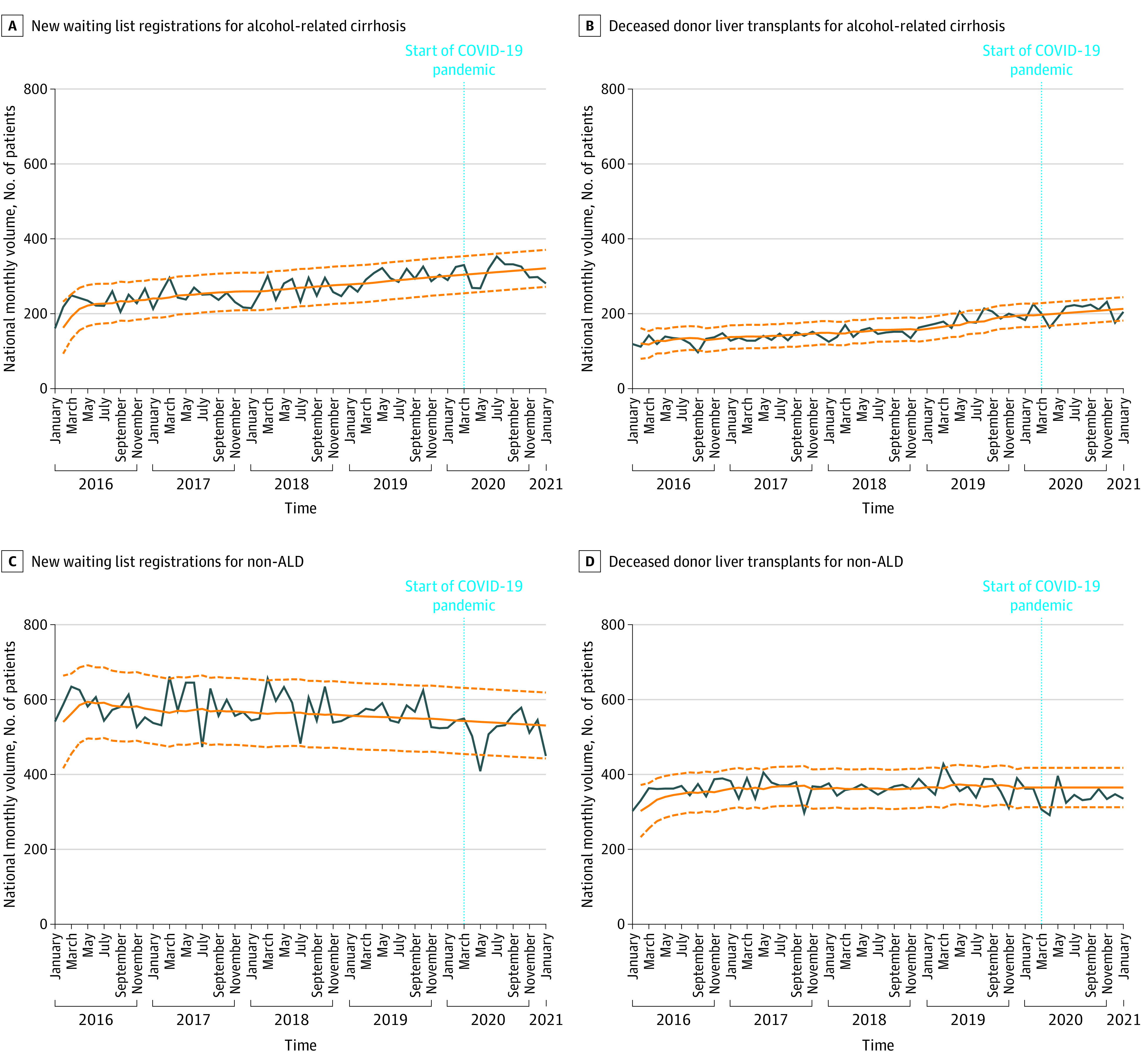
Observed and Forecasted National Monthly Volumes of New Waiting List Registrations and Deceased Donor Liver Transplants Observed values are shown in blue. Historical trends and forecasted values (solid line) in the absence of COVID-19 with 95% CIs (dashed line) are shown in orange. ALD indicates alcohol-associated liver disease.

A total of 51 488 new waiting list registrations and 32 320 DDLTs from January 1, 2016, to January 31, 2021, were assessed. The median (IQR) age was 58.0 (50-64) years among pre–COVID-19 waiting list registrants and liver transplant recipients. The median (IQR) age was 58.0 (49-64) years for new waiting list registrants during COVID-19 and 58.0 (48-64) years for transplant recipients during COVID-19. Among new waiting list registrants, 15 247 (36.1%) were women and 26 930 (63.9%) were men during the pre–COVID-19 period, and 3477 (37.3%) were women and 5834 (62.7%) were men during COVID-19 (*P* = .03). From March 2020 to January 2021, during COVID-19, there was a significant increase in the proportions of waiting list registrations (227 of 9311 registrations [2.4%] vs 138 of 9638 registrations [1.4%]; *P* < .001) and DDLTs (185 of 6162 DDLTs [3.0%] vs 103 of 6263 DDLTs [1.6%]; *P* < .001) for AH compared with the same period pre–COVID-19 (ie, March 2019 to January 2020). Compared with long-term pre–COVID-19 trends, there was an overall reduction in waiting list registration and DDLT at the start of COVID-19, from March to May 2020 (Figure 1A and B).^5^ From June 2020 to January 2021, observed volumes of waiting list registrations and DDLTs for AH increased sharply, exceeding forecasted values by a mean of 59.5% in waiting list registrations and 62.0% in DDLTs and surpassing the 95% CI for 4 of 8 months. There was a temporal association and positive correlation between increasing waiting list registrations (Spearman ρ = 0.79; *P* < .001) and DDLT (Spearman ρ = 0.76; *P* < .001) for AH and increasing retail alcohol sales (Figure 1C). Observed waiting list registrations and DDLTs for AC and non-ALD remained within or below forecasted values (Figure 2).

## Discussion

This cross-sectional study found that waiting list registrations and DDLTs for AH increased significantly during COVID-19, exceeding the volumes forecasted by pre–COVID-19 trends by more than 50%, whereas trends for AC and non-ALD remained unchanged. While we cannot confirm causality, this disproportionate increase in association with increasing alcohol sales may indicate a relationship with known increases in alcohol misuse during COVID-19.^1^ Since less than 6% of patients with severe AH are listed for transplantation, increasing waiting list volume during COVID-19 represents a small fraction of the increase in AH, a preventable disease with 6-month mortality up to 70%.^6^

Our study is limited by its retrospective nature and changes in allocation policy and transplant center practices that may have independently affected waiting list registration and DDLT. Although allocation changes in February 2020 could have contributed to increasing DDLT for AH, the simultaneous increase in waiting list registration supports an underlying increase in AH. While increasing adoption of early DDLT for AH has increased waiting list registration and DDLT for AH in recent years,^2,6^ this would not explain the acute increase during COVID-19 disproportionate to pre–COVID-19 trends. This study provides evidence for an alarming increase in AH associated with increasing alcohol misuse during COVID-19 and highlights the need for public health interventions around excessive alcohol consumption.
